# Family Conversations About Heat and Temperature: Implications for Children’s Learning

**DOI:** 10.3389/fpsyg.2020.01718

**Published:** 2020-08-18

**Authors:** Megan R. Luce, Maureen A. Callanan

**Affiliations:** Department of Psychology, University of California, Santa Cruz, Santa Cruz, CA, United States

**Keywords:** science talk, parent–child communication, conceptual change, scientific thinking, sociocultural perspectives

## Abstract

Some science educators claim that children enter science classrooms with a conception of heat considered by physicists to be incorrect and speculate that “misconceptions” may result from the way heat is talked about in everyday language (e.g., Lautrey and Mazens, 2004; Slotta and Chi, 2006). We investigated talk about heat in naturalistic conversation to explore the claim that children often hear heat discussed as a substance rather than as a process, potentially hindering later learning of heat as energy involved in emergent processes. We explored naturalistic speech among children and adults to understand the nature and the frequency of heat- and temperature-related conversations that young children are involved in. This study aims to investigate the actual linguistic resources that children have available as part of a sociocultural approach to cognitive development. Parents’ everyday conversations about heat and temperature with their 2–6-year-old children were drawn from the Child Language Data Exchange System (CHILDES) language database and from a parent–child book-reading study. Parents used the word *heat* rarely, but they did so in ways that implied it is a substance. Parents never talked about heat as an emergent process but sometimes as a direct causal process. Most of the heat- and temperature-related talk, however, focused on words like *hot* and *cold* to describe temperature as a property of objects. This investigation of what young children actually experience in everyday conversations is a step toward studying how everyday language may play a role in children’s understanding of heat and temperature.

## Introduction

Children develop “intuitive” ideas about key physical concepts and phenomena through everyday navigation of their environments and activities (Piaget, 1974; diSessa, 1996; Wellman and Gelman, 1998; Gopnik and Meltzoff, 2002; Wilkening and Huber, 2004), and yet paradoxically, they often have great difficulty understanding similar concepts later in science classrooms (e.g., McCloskey, 1983). Debates about this discrepancy between “naïve” physics and formal physics have often pitted theories that emphasize the cognitive aspects of conceptual change against theories that emphasize the processes of reasoning occurring in the context of phenomenological experience or sociocultural activities. A more productive path may be to acknowledge that individuals have multiple repertoires (both conceptual and experiential) for understanding complex scientific concepts (Hammer, 2000; Amin, 2009, 2015; Vosniadou, 2009). In this study, we draw upon a sociocultural framework by focusing on a crucial piece that is often left out of these discussions: a characterization of the everyday experiences within which children come to understand and use such concepts (Rogoff et al., 2018).

Heat and temperature provide an excellent example of a conceptual domain where children’s well-developed everyday notions come up against very different scientific notions. For example, toddlers begin to learn about temperature concepts by experiencing and talking about such things as cold water, hot stoves, and warm fuzzy slippers. Despite extensive evidence of young children’s clear expression of temperature concepts in everyday language, understanding the concepts of heat and temperature in formal science instruction proves to be difficult for many students even in high school (e.g., Clough and Driver, 1985; Erickson and Tiberghien, 1985; Lewis and Linn, 1994). Some science education researchers have suggested that this results from “misconceptions” in young children’s everyday knowledge that are often quite distinct from current “scientific” conceptions of these phenomena (Shtulman, 2017; see Abimbola, 1988; Hammer and Van Zee, 2006, for discussions of the term “misconception”). For example, there is evidence that students often conflate the concepts of heat and temperature (Wiser and Carey, 1983; Wiser, 1995). Chi and colleagues (Slotta et al., 1995; Chi, 2005) argue that students incorrectly conceptualize heat as a substance, which leads to a difficulty in understanding heat as an emergent process.

Prior research and theory suggest the importance of two essential sources of children’s learning of concepts such as heat and temperature. First, scientific concepts are argued to emerge in children’s phenomenological experiences (diSessa, 1993b). Second, instruction (Chi, 2005), also called “testimony” (Harris, 2002; Harris and Koenig, 2006) or “input” (Gelman, 2009), is argued to serve as another distinct source for children’s conceptions. Taking a sociocultural perspective, our view is that these two sources are, in fact, intertwined in the learning that happens in children’s everyday lives, where testimony occurs in the midst of phenomenological experience (Rogoff et al., 2018). Several researchers have speculated that everyday language about heat and temperature may mislead children to ideas about these topics that differ from the current scientific ideas. For example, Slotta and Chi (2006) speculate that the ways heat is talked about in everyday contexts may contribute to the miscategorization of heat as a substance rather than as an emergent process. Furthermore, Lautrey and Mazens (2004) suggest that there are few linguistic supports in everyday language for thinking about heat as energy transfer (in contrast to sound, also argued to be an emergent process, for which terms such as *resonate* and *vibrate* are available to intuitively describe it). Despite these speculations, there is very little systematic investigation of the actual everyday linguistic supports for thinking about heat and temperature that are available to young children, either in family settings or in classroom settings. Rather, most of the previous research assesses children’s understanding in structured experimental settings (e.g., Wiser and Amin, 2001; Clark and Jorde, 2004). Our goal in this study is to illuminate the potential contribution of spontaneous everyday language during family activity to children’s developing conceptions of heat and temperature. Along with others (Rosebery et al., 2010), we argue that children’s experiences, interpretations, and meanings of the phenomena of temperature are varied. Therefore, we cannot assume that all children have similar experiences and meanings to draw upon in any given context. In this study, we aim to explore the variation in linguistic contexts in which children converse with others about temperature in order to expand our knowledge of variation and its potential importance in science learning.

As background to the current study, we first present Slotta and Chi’s ontological framework for understanding children’s concepts of heat. Second, we briefly discuss the alternative views raised by others regarding both children’s concepts and scientists’ concepts of heat, considering both diSessa’s “knowledge-in-pieces” view and the “theory theory” view. Finally, we argue that investigation of parent–child conversations in meaningful activity is necessary to fully understand how children’s scientific knowledge develops (Rogoff, 1990; Callanan and Jipson, 2001; Crowley and Jacobs, 2002; Rogoff et al., 2018) and, in this case, to evaluate the claim that everyday language encourages particular misunderstandings. We argue that instead of pitting theories against each other, we can find productive points of overlap that open up new avenues for research.

### Slotta and Chi’s Ontological View

Slotta, Chi, and colleagues (Slotta et al., 1995; Reiner et al., 2000; Chi, 2005; Slotta and Chi, 2006) proposed that students’ difficulty in understanding the nature of heat stems from their misconceptualization of heat as a substance rather than as a process. Drawing on developmental psychology literature (e.g., Keil, 1979, 1983), they have claimed that students incorrectly assign heat to the ontological category of substances. They argue that heat is best conceptualized not as a substance but as an “emergent process,” defined as dynamic ongoing interactions of the components within a system, which involves energy transfer and simultaneity at a molecular level (e.g., diffusion of two liquids). More recently, Chi and colleagues have argued that when students do think of heat as a process, rather than as a substance, they still usually misconstrue it as a “direct causal process.” In contrast to emergent processes, direct causal processes are defined as simple causal interactions in which the behaviors of the components within a system are distinct and sequential (e.g., a heart pumps, causing blood to circulate). Some argue that the emergent process view of heat is used or preferred by physicists (Slotta et al., 1995; Slotta and Chi, 2006; but see Zemansky, 1970; diSessa, 1993a; Hobson, 1995, for other process views). In one study, physics novices (9th graders) and physics experts (graduate students and postdocs) responded to heat-related problems (e.g., “Two cups of hot coffee are poured into two cups: a Styrofoam cup and a ceramic mug. Both are sealed with airtight lids. After 20 min, what would you predict for the temperatures in both cups?”) The participants’ language was coded as substance-based if they expressed, for example, that heat gets trapped (contained) or that it moves from object to object, and as emergent process-based if they talked about heat as a process of energy transfer (including excitation of molecules). Physics novices were more likely to use substance predicates than process predicates for the heat problem, whereas physics experts were more likely to use process predicates (Slotta et al., 1995).

Children and non-expert adults in Slotta and Chi’s studies responded in ways that suggest they think of heat as a substance or direct causal process rather than emergent process (Slotta et al., 1995; Reiner et al., 2000; Slotta and Chi, 2006). In support of this view, earlier interview research found that young children (under 6 years) seemed to view heat and cold as distinct substances that are inherent properties of objects and that can be accumulated and contained (Albert, 1978; Erickson, 1979; Clough and Driver, 1985). The oldest children studied (9–10-years-old) mentioned concepts of heat energy transfer similar to conduction (energy transfer due to contact) and to radiation or convection (energy transfer by proximity or currents); however, it is unclear whether these children understood heat as an emergent process.

Chi and colleagues argue that because students do not readily understand the class of emergent processes, they must be instructed to learn this ontological category and reassign the concept of heat to it (Slotta and Chi, 2006). In various studies, they measured which ontology (substance or emergent process) high school and college students used to assign the concept of heat (and other phenomena such as light and electric current) before and after instruction in the emergent process ontology (Slotta et al., 1995; Slotta and Chi, 2006). In one study, instructing undergraduate students in the emergent process ontology facilitated their greater use of emergent process predicates on problems of electric current when compared to a control group (Slotta and Chi, 2006). Wiser and Amin (2001) provided some preliminary evidence for the potential effectiveness of such instruction. Four eighth-grade students were directly instructed that there are two alternative ways to think about heat and temperature: an “everyday” and a “scientific” way. Students were explicitly taught that these ways of thinking make use of the same words (e.g., heat) but convey different meanings (scientific *heat* refers to a process of energy transfer, whereas everyday *heat* can refer to a substance that moves). Children who participated in this metaconceptual teaching unit were argued to have come to view these as compatible but differentiated models based on their tendency to talk about heat as molecular energy when solving problems in a testing situation after the lessons, where the interviewer asked them to clarify their statements in terms of “everyday” and “scientific” heat.

### Alternative Views: Knowledge in Pieces and Theory Change

The distinction Slotta and Chi made between substance-based and emergent process-based concepts of heat has been questioned from the standpoint of how both children and practicing physicists think about heat. diSessa (1993a) suggested that people do have everyday experiences with more physics-like ontologies, such as equilibrium changes, and thus may have some intuitive knowledge about processes. He further questions the assumption that most physicists attribute heat, as a form of energy, to an emergent process ontology, arguing instead that physicists may think about energy as a substance-like entity (having location, being conserved, “flowing,” even having mass) that is transferred in situations such as heating. In fact, many physicists may, at times, find it appropriate to speak about heat as a substance, as indicated in some physics textbooks (e.g., ter Haar and Wergeland, 1960; Reif, 1965). For example, Reif (1965) mentions that the “amount of heat is transferred” (p. 106) and “heat is absorbed by the system at the lower absolute temperature *T* and given off by the system.” (p. 106). Granting that this language is likely being used in a metaphorical way or as a strategy to avoid the difficulty of explaining the complex non-intuitive notion of emergent processes, the variability in children’s, as well as physicists’, thinking (or at least ways of expressing concepts) still suggests that knowledge about heat may not be organized strictly in terms of ontological categories such as substance and process (diSessa, 1993b; Clark, 2006; Siegler, 2007). Indeed Chi later revised her claim to include conceptualizations of heat as “direct causal process” as more advanced than a substance-based view, although still inferior to an emergent process view.

Theory change proponents, rather than focusing on children’s misconceptions, argue that conceptual change occurs through the gradual formation and the subsequent revision of broad intuitive theoretical frameworks (see Carey, 1985, 1991; Vosniadou and Brewer, 1992; Gopnik and Meltzoff, 2002; Lautrey and Mazens, 2004). While children’s statements often seem inaccurate in light of the adult or scientific view, they are argued to be consistent with a coherent alternative theory held by the child. For example, children may first have a theory that conflates heat and temperature, and only later in development separate those into two distinct concepts. Also, children may revise components of theories at different rates, resulting in the differential understanding of two phenomena – sound and heat, for example (Lautrey and Mazens, 2004) – supporting the notion that children can hold distinct theories that can be revised at different rates based on evidence.

Proponents of the knowledge-in-pieces view argue that knowledge about physics is more loosely organized than either ontological categories or theories, such that children, as well as physicists, do not have a single conception of heat or may necessarily use several ontologies when thinking about heat (diSessa, 1988, 1993a; Gupta et al., 2010). On this view, the expression of knowledge may appear as assemblies of pieces coming together in the moment to solve a particular problem or as a process of drawing upon various resources one has for thinking about a phenomenon (Hammer, 2000). Findings that are argued to support this view include those showing that people are likely to reason differently across contexts (Clark and Jorde, 2004; Clark, 2006) and that thinking about varied prior experiences can help students engage with different aspects of a phenomenon (Rosebery et al., 2010). For example, when responding to heat-related word problems, eighth-grade students often expressed multiple contradictory ideas, which included claiming at one point that metal objects will be of the same temperature as glass objects in an oven and claiming at another point that the metal objects will be hotter because metal is a good conductor (Clark, 2006). Children also reasoned differently depending on the context of the task and used prior experiences to support their reasoning. For example, one student claimed that wood and metal objects would be of the same temperature when placed in a hot trunk but not when placed in a hot oven. Others claimed that metal is a good insulator because people wrap soda cans in aluminum foil to keep them cold (see also Lewis and Linn, 1994; Brookes and Etkina, 2015).

The literature presents conflicting results regarding how children’s knowledge is organized and how learning occurs, and multiple theories have different explanations for the same findings. The available evidence has been argued to support the ontological category view, the theory theory view, and the knowledge-in-pieces view (diSessa, 1988; Lautrey and Mazens, 2004; Slotta and Chi, 2006), and yet theorists on all sides of the debate have competing explanations for similar data. For example, while some theorists see inconsistent responses about heat as evidence of “knowledge in pieces,” others argue that they are evidence that children are in transition between two coherent theories (Lautrey and Mazens, 2004) or that children have a coherent but incomplete version of an ontological view (Chi, 2005).

Studies of children’s conceptions of phenomena such as heat and sound (e.g., Mazens and Lautrey, 2003; Clark, 2006) have aimed to test a theory theory view against a “knowledge-in-pieces” view. By using semi-structured interviews to assess 6–10-years-old children’s predictions and explanations for sound-related problems, Mazens and Lautrey (2003) found that children were not consistent in interpreting sound as a substance. Children instead assigned some aspects of sound to process ontology, suggesting that they entertain multiple ontologies for sound at the same time. Mazens and Lautrey (2003) interpreted this as evidence for theory theory and as evidence for the gradual transition from one intuitive theory to another. However, one might argue instead that such variation in the same child’s responses could support a more flexible view of knowledge, such as the knowledge-in-pieces view (Gupta et al., 2010). Furthermore, Slotta and Chi (2006) revised their claim that the substance ontology must be replaced by an emergent process ontology, suggesting that these two ontological categories can exist in parallel, where students learn which one is appropriate in which situations. Thus, it is difficult to empirically distinguish among these three theoretical approaches. Indeed more recent work argues for developing a new view that moves beyond trying to choose among theories and instead considers how learners incorporate multiple types of resources (including embodied experience, conceptual metaphors and other cognitive models, scientific language and everyday language) in understanding abstract scientific concepts (Hammer, 2000; Amin, 2015).

In order to integrate the sources available to the child, one type of data that is largely missing is the detailed analysis of young children’s exposure to ideas about heat and temperature as they relate to everyday discourse. Regardless of one’s theoretical orientation, we suggest that a crucial next step is to investigate the varied everyday situations in which young children experience talk and action related to heat and temperature. In particular, we need more information about everyday discourse in order to articulate the data available to children as they create and revise their understanding of heat and temperature across contexts.

### Social Context of Scientific Thinking

From each of the outlined perspectives – ontological, knowledge-in-pieces, theory change – the way heat is talked about in everyday contexts is a potentially important piece of the puzzle regarding how children come to understand heat and respond to heat-related problems across contexts. Slotta and Chi and others speculated that commitments to ontologies such as substance may be reinforced by everyday language (Zemansky, 1970; Erickson and Tiberghien, 1985; Hobson, 1995; Slotta and Chi, 2006), and recent data support some of these claims (Brookes and Etkina, 2015). For example, someone might say: “Shut the door, you’re letting out all of the heat.” Hearing *heat* used as a noun with the implication that it is a substance that can move from one location to another may present an obstacle to understanding heat as energy or as an emergent process.

While diSessa (2000) and others would disagree that speaking about heat as if it is a substance or a direct causal process is categorically limiting or wrong, the knowledge-in-pieces view also calls for an account of how children encounter discourse about heat and temperature in everyday situations. diSessa’s view focuses mostly on the phenomenological experiences of heat and temperature, but we suspect that he would agree that it is important to investigate how language is used to describe or make sense of those experiences as well. diSessa (2000) describes the potential disconnect between learning the words and the meanings of a physical phenomenon and how that phenomenon is experienced. Related to this point, a recent study by Jeppson et al. (2017) used infrared cameras to support 7–8-year-old students in discussing and conceptualizing heat as a process in the context of their embodied experience. Thus, it seems important to investigate how students merge experiences of heat and temperature with words and meanings that people use to communicate about them (see also Rosebery et al., 2010).

Finally, from a theory theory perspective, Lautrey and Mazens (2004) argue that children’s theories of heat and sound may be partly influenced by ways that language does or does not provide support for thinking about such difficult concepts. For example, hearing the expressions *vibrate* and *resonate* for the process of sound may provide children with a “direct intuition” about the mechanism of sound transfer. They suggest that language supports for heat transfer seem less available than those for sound in everyday experiences. This points to the importance of considering everyday experiences in children’s theory revision processes.

We argue, then, that what is sorely needed in the field is an analysis of the language that young children actually hear from their parents (and other adults) about heat and the related concept of temperature. Do parents talk about heat in ways that suggest that heat is an entity or a substance that moves from one object to another? How do parents and children talk about the related concept of temperature? Parent–child conversation is a prominent social interaction context for young children. Research on Western parent–child conversations reveals that such contexts can offer rich opportunities for conceptual development and scientific thinking in which parents help children understand the world as they experience it and offer knowledge about the world to which children may not have direct access (Vygotsky, 1978; Bruner, 1983; Tizard and Hughes, 1984; Callanan and Oakes, 1992; Snow and Kurland, 1996; Crowley et al., 2001; Harris, 2002). Investigating the linguistic context in which children learn about heat and temperature would further allow for future analysis of the links between language and non-linguistic conceptualizations of heat. We can also ask about whether parents’ talk about heat and temperature varied as a function of children’s age within the age range from toddlers to school entry. It is possible that parents’ language may change over this age range as they perceive children to become more sophisticated in their comprehension.

The present study investigates children’s early experiences with heat- and temperature-related concepts in several corpora of naturalistic parent–child conversations (three cross-sectional and three longitudinal data sets). Conversations were drawn from the Child Language Data Exchange System (CHILDES) and from a study of video-recorded book-reading sessions (Callanan et al., 1995) to explore the range of everyday contexts in which parents and children talk about heat and temperature.

## Materials and Methods

### CHILDES Database

We used the CHILDES as a source of naturalistic family conversation about heat and temperature. Created by Brian MacWhinney (1995), CHILDES is a shared database of transcripts and audio files of children’s recorded conversation and speech. The corpus consists of Institutional Review Board-approved data that are contributed by researchers. For this study, British English conversations were excluded because of our unfamiliarity with the dialect. Four criteria were used to select databases to include in this study: The children studied were between the ages of 2 and 7 years, the recordings of speech were in everyday settings such as homes, parents were present during the recordings, and the database included only one study (some databases include transcripts from multiple studies with different samples of children and different settings).

#### Participants

The CHILDES data were drawn from five databases: Hall (Hall and Tirre, 1979), Gleason (Gleason et al., 1984), Sachs (Sachs, 1983), Kuczaj (Kuczaj, 1976), and Brown (Brown, 1973). Table 1 displays information about the nature of the data collection, demographic information, age, and gender for the participants.

**TABLE 1:** Participant information for each data source.

Data source	Data collection	Demographics	Age and gender
**CHILDES**			
Hall	Study of vocabulary use in children from various socio-economic and racial groups. Families observed at home, school, and in route to school.	39 children sampled from 4 populations: Black working-class White working-class Black middle-class White middle-class	4;6–5;0 Gender N/A (roughly equal boys and girls)
Gleason	Study of acquisition of communicative competence. Dinnertime conversations between children, mothers, and fathers in their homes.	22 children White, middle-class Boston, MA area	2;0–5;2 11 girls 11 boys
Sachs	Researcher collected speech samples from her daughter in her home. Longitudinal data. (Naomi)	1 child	1;2–5;1 girl
Kuczaj	Researcher collected speech samples from his son in his home. Longitudinal data. (Abe)	1 child	2;4–5;0 boy
Brown			
Adam	Speech collected from child in his home.	1 child Middle-class, Black family	2;3–4;10 boy
Eve	Speech collected from child in her home.	1 child Middle-class White family	1;6–2;3 girl
Sarah	Speech collected from child in her home.	1 child Working-class White family	2;3–5;1 girl
**Snowman Study**	Videorecorded interactions of parents and children reading a book together in their home.	51 children Mostly middle- to upper-middle-class White families.	2;0–5;3 25 girls 26 boys
			

#### Materials and Procedure

The speech recordings were transcribed in the CHAT transcription format and uploaded to the CHILDES database by each contributor (MacWhinney, 1995). We used the CLAN program (*kwal* command), designed for searching and analyzing transcript information, to search the transcripts for the heat- and temperature-related words shown in Table 2. The keyword search was broadened beyond the term *heat* because it was predicted that parents and children might use many other words conceptually related to heat, such as *hot*, *cold*, and *warm*.

**TABLE 2:** The heat- and temperature-related words (and frequency of occurrence) searched for in the Child Language Data Exchange System databases and Snowman Study transcripts.

Cold (931)	Cooler (2)	Hotter (7)	Warmest (0)
Colder (5)	Cooling (2)	Hottest (0)	Warming (3)
Coldest (4)	Heat (42)	Warm (257)	Warms (3)
Cool (126)	Heated (4)	Warmed (8)	
Cooled (8)	Hot (818)	Warmer (14)	

The utterance that contained the keyword and the three lines before and after the utterance were returned in CHAT format. These seven-line conversation segments were then coded. The entire transcript was consulted if more conversational context was necessary to interpret the utterance. Examples of seven-line conversation segments are shown below:

Keyword: hot. File “gas.cha”: line 14452
Mother: you observe more than I do #.
Child: what is dis [: this] black stuff?
Mother: that’s just the cheese browning.
**Mother: when the oven is hot, cheese browns a little bit**.
Mother: it taste good.
Mother: sure you don’t want a taste #?
Child: you please don’t have any of moi [: my] stuff.
Keyword: warm. File “ded.cha”: line 690
Father: it’s not time yet.
Father: it’s not time to go yet.
Child: yeah well I’m getting in [!].
**Father: no, because you will be too warm in this inside.**
Child: no I won’t.
Father: wait Mommy still has a little more time left, you’re gonna [: going to] be too warm and then you’ll go out and get a cold.
Father: right?
Keyword: warm. File “adam03.cha”: line 1495
Child: Adam don’t wear wear shoe.
Mother: yes # Adam does wear shoes.
Child: take shoe off?
**Mother: shoes help keep your feet warm.**
Child: keep feet warm?
Mother: what do you wear over your shoes when it’s raining?
Child: oh no xxx wear shoes.

#### Coding Heat and Temperature Words

A five-category coding scheme was developed to capture the ways that parents and children talked about the word *heat* and other temperature words: *substance*, *emergent process*, *vague process*, *property*, and *other meaning*. Table 3 shows a list of the coding categories and examples from the data. The coding scheme began with the original substance and emergent process codes^1^ of Slotta et al. (1995), and other codes were added.

**TABLE 3:** Heat- and temperature-related coding categories and examples from the data.

Type of talk	Examples
Substance	We don’t have that kind of heat. So it must be very sensitive to heat. Child: I see fire. Parent: What about the heat? He likes the cold.
Emergent process	The stove transferred the heat to the pot. (Slotta et al., 1995)
Vague process	I’m getting warmer. Okay, I’ll put the roast back in to keep it warm. To heat up the house.
Proximity	(he’s too near the fire) he’s going to get too hot. You getting hot in here?
Time	Let it warm up first. Oh, it’s cool now?
Contact/Presence	That’s a jacket to keep me warm. The sun makes you hot, right?
Property	Child: It’s cold. Parent: What’s cold? Your milk? He’s hot but I’m cold. The sun’s hot, right.
Other	Did you catch a cold? I’m hot on his trail.

The *substance* code was used to identify cases in which heat or heat- and temperature-related ideas were expressed in substance predicates, implying that heat can be contained, quantified, absorbed, and accumulated (e.g., “You can’t feel the heat?” “The heat…is out in the room,” “Heat right in the fire,” “Close that window, it’s cold in there we got no heat”).

The *emergent process* code was used to identify cases in which heat or heat- and temperature-related ideas were expressed with the particular process predicates that Slotta et al. (1995) identify as reflecting a conceptualization of heat as an acausal interaction or a constraint-based interaction. Emergent process predicates imply that heat is a process involving transfer, excitation, interaction, equilibrium-seeking, simultaneity, and uniform and continuous interactions of components (Chi, 2005; see Table 3 for an example).

The *vague process* category was added because parents and children sometimes identified temperature change as a process, without including the word heat. They discussed temperature change in ways that did not reflect an emergent process conceptualization (at the molecular level) and instead focused on processes at a more macroscopic level. Vague process includes mentions of the heating or temperature change process without indicating heat as a substance (e.g., “Just heat it and serve it,” “We have to wait for it to heat up,” “It’s getting warmer,” “You don’t want to get cold”), suggesting that heating and cooling are macro-level processes. References to temperature change were also further coded into three sub-categories: *proximity* indicated that temperature change is related to whether an object is close to or far from a heat source or indicated the location of an object, *time* included explicit mentions of time, and *contact* indicated that temperature change is associated with coming into contact with or being in the presence of a heat source (see Table 3).

In addition to the substance and process codes, a *property* code was developed to capture talk about heat and temperature words as adjectives, suggesting that they are properties of objects (e.g., “That’s a hot fire,” “My peas are cold”).

The *other meaning* category was included to capture metaphorical and other uses of hot and cold as well as isolated words that did not have sufficient context to determine the intended meaning (see Table 3).

The utterance that contained the keyword was the unit of analysis, and utterances that contained more than one keyword were coded only once. For example, the utterance *this is hot and that is cold* was given only the property code. This was done to keep the coding unit at the utterance level already determined in the CHAT transcription guidelines. For utterances that contained two keywords that could be coded differently, the primary coder determined which code best captured the meaning of the conversation within which the utterance was embedded. Inter-coder reliability was established on 20% of the utterances. Percent agreement ranged from 85 to 89% and Cohen’s Kappas ranged from 0.75 to 0.80 (excellent level of agreement according to Bakeman and Gottman, 1986). Disagreements were resolved through discussion.

#### Coding Conversational Contexts

A nine-category coding scheme was developed to capture the range of conversational contexts in which parents and children talked about aspects of heat and temperature. Each of the 1,738 heat- and temperature-related utterances in our selected CHILDES data was coded into one of the nine categories. The categories aim to capture context in a broad sense that considers both activity setting and topic discussed, including either participating in or talking about meal times, weather, body temperatures, dressing in clothing, touching objects, heat sources, bathing, and swimming. A ninth category (other) included the idiomatic uses of the keywords as well as cases where the context was not clear from the transcript. To establish inter-coder reliability, two people coded 33% of the utterances. Cohen’s Kappa was 0.95 (excellent agreement). Disagreements were resolved through discussion.

### Snowman Book-Reading Task

#### Participants

Fifty-one parent–child dyads participated in this book-reading study (see Callanan et al., 1995), including four age groups: 2-year-olds (six boys and six girls, mean age = 23.87 months, *SD* = 1.70, range = 22–27 months), 3-year-olds (seven boys and seven girls, mean age = 35.71 months, *SD* = 1.43, range = 33–38 months), 4-year-olds (six boys and six girls, mean age = 45.58 months, *SD* = 2.71, range = 42–50 months), and 5-year-olds (seven boys and six girls, mean age = 58.46 months, *SD* = 1.94, range = 56–63 months). Of the 51 parents, 47 were mothers and four were fathers. The families were recruited through daycare centers and informal contacts of the researchers and other participants (see Table 1 for data collection and demographic information).

#### Materials and Procedure

Parents and children read the wordless children’s book *The Snowman* (Briggs, 1978). The storybook depicts a boy’s adventure in making a snowman that “comes to life” and is introduced to household items such as a fireplace, stove, hot water, and a freezer. Because the book does not contain words, parents and children may narrate the story as they wish. It was expected that because of the snowman’s encounters with hot and cold household items and his propensity to melt, parents and children would talk about heat and temperature.

The researchers video-recorded the families in their homes. The parent–child dyads (one parent and one child) first engaged in making muffins together, with the exception of the 2-year-olds who only read the book. While the muffins were baking, the parent and the child read the book together. The video-recorded sessions were transcribed.

#### Coding

The transcripts were searched for temperature-related words (see Table 2) as in the CHILDES procedure. The five-category coding scheme (substance, emergent process, vague process, property, and other) described in the CHILDES coding section above and in Table 3 was also used for the Snowman Study analysis. Inter-coder reliability was established on 20% of the utterances. Percent agreement was 89–91% and Cohen’s Kappa was 0.83 (excellent; Bakeman and Gottman, 1986). Disagreements were resolved through discussion. Because in this data set each parent-child dyad was engaged in the activity of book reading, the conversation context coding scheme was not relevant.

## Results and Discussion

This section is divided into three main analyses (with discussion following each analysis) that include both the CHILDES and the Snowman Study, and main findings are provided and discussed for each analysis. Analysis 1 tests the claim that children hear adults talk about *heat* as a substance. Analysis 2 explores the range of different heat- and temperature-related words parents and children used, asking how often they occurred in everyday conversations, and how often they were used as property, substance, or process. Analysis 3 describes how such words were used across different everyday activity contexts for the CHILDES databases only.

### Analysis 1: The Keyword Heat Only

Because the word *heat* is of particular importance in conceptions of heat and temperature, it was separated out from all other keywords for analysis. This analysis tested the claim that young children often hear the word *heat* talked about as a substance (Slotta and Chi, 2006).

#### Main Finding 1

Parents used the word *heat* rarely, but when they did, they most often talked about heat in ways that are consistent with it being a substance.

Of the 2,138 coded utterances across all CHILDES databases and the Snowman Study data, 49 contained the keyword *heat*. Of these instances, 42 (86%) were spoken by parents and seven (14%) were spoken by children. For children, three (43%) of their references to heat were coded as substance, three (43%) as vague process, and one (14%) as other. For parents, 29 (69%) of their references to heat were coded as substance and 13 (31%) were coded as vague process [χ^2^(1, *N* = 42) = 6.10, *p* = 0.014]. As Chi and colleagues would predict, emergent processes were never discussed.

As an exploratory analysis, we developed a sub-category of the substance code (substance-involved-in-direct-process) that was used to identify the instances when parents or children seemed to identify heat as a substance but also implied that it was involved in a direct causal process where, based on Chi’s conceptualization, there is directionality and the movement is not described explicitly as continuous or simultaneous (e.g., “This is where the heat comes in this house,” “And they’re still sending up the heat,” “You hold it in your hands and it gets soft so that you get the heat from your hands”)^2^. The percent agreement between two coders was 88%; Cohen’s Kappa = 0.80 for all *heat* statements.

Further analysis including the substance-involved-in-direct-process code made little difference in the pattern of children’s talk; of their three substance comments, one was recoded as substance-involved-in-direct-process. For parents, 20 of their 29 references to heat remained coded as substance, and nine were re-coded as substance-involved-in-direct-process. Thus, a strict interpretation of Chi’s idea is supported – parents do most often talk about heat in ways that are consistent with it being a substance and never as an emergent process. Children were also heard talking about heat involved in macro-level processes. Almost equal in frequency to the talk about heat as a substance were the comments about heat as a general process, if we combine substance-involved-in-direct-process and vague process talk compared with substance talk (52 versus 48%). However, it is important to emphasize that this is very different from emergent process at the molecular level. When parents used the word *heat*, then it was most often either substance-based or focused on direct (macro-level) causal processes. At the same time, the word *heat* occurred rarely in these conversations, which suggests that children may actually be getting very few opportunities to hear about how adults conceptualize the concept of heat in everyday talk. In the few instances where children used the word *heat*, they were equally likely to use substance-based language and vague process-based language.

Most of the conversations about heat and temperature did not include the word *heat*. The next analysis focuses on utterances that contained the other temperature-related words.

### Analysis 2: All Temperature Keywords (Excluding Heat)

*Cold* and *hot* were the most frequently observed words (see Table 2). Table 4 displays the percentage of utterances in each database containing temperature words that were coded as each type of talk. Heat- and temperature-related words were never talked about as emergent processes in any CHILDES database or in the Snowman Study; therefore, the category was excluded from analyses. Because the metaphorical or other meanings of heat- and temperature-related words were not of interest for the current study, the other category was also excluded from subsequent analyses. In databases where children’s gender was known, analyses were initially conducted with gender as a between-subjects factor. No significant effects or interactions involving gender were found; therefore, it was excluded from all subsequent analyses. All analyses were conducted separately for parents and children.

**TABLE 4:** Percentage of “heat” and temperature utterances coded as each type of talk by database.

	Abe	Adam	Eve	Gleason	Hall	Naomi	Sarah	Snowman
Utterances (n)	289	127	130	127	592	155	318	400
Property	78%	66%	68%	83%	68%	78%	76%	69%
Substance	< 1%	< 1%	6%	0	3%	1%	4%	5%
Emergent	0	0	0	0	0	0	0	0
Process								
Vague	19%	17%	23%	14%	18%	12%	8%	24%
Process								
Other	4%	17%	2%	3%	10%	8%	12%	3%

In analysis 2, our second and third main findings are stated and then the corresponding analyses from each of the relevant databases are presented.

#### Main Finding 2

Both parents and children talked about heat- and temperature-related words as properties of objects more often than they talked about them as vague processes or as substances in all but one of the databases. The only exception is that parents of older children in one database (Gleason) talked equally about properties and vague processes.

#### Main Finding 3

Although the parents did not talk in ways that are consistent with emergent processes or substances, they did sometimes give information about vague processes. In addition, parents in the Snowman Study mentioned three aspects of the process of temperature change.

#### CHILDES Database: Gleason Dinner

**FIGURE 1:**
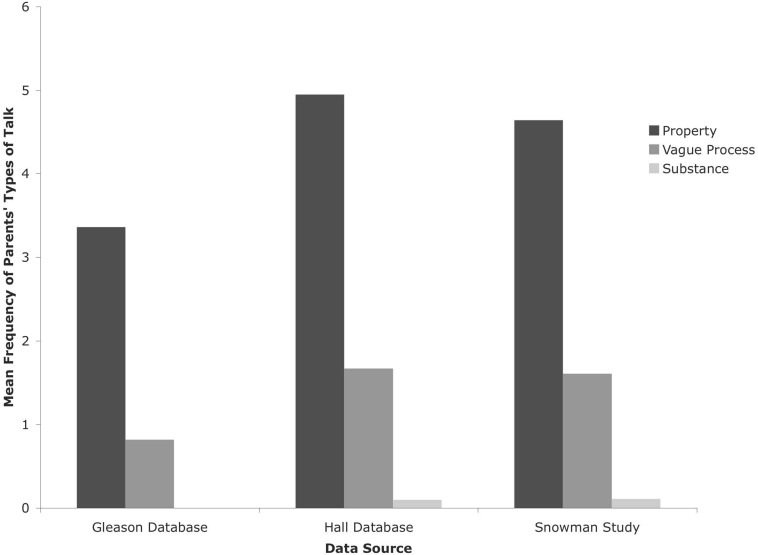
Types of Talk for parents from Gleason, Hall, and Snowman Study.

In this database, neither parents nor children talked about temperature-related words as substances. Therefore, a paired-samples *t*-test compared the parents’ use of two types of talk: property and vague process. As shown in Figure 1, the parents talked about temperature-related words as properties of objects (*M* = 3.36, *SD* = 5.57) more frequently than they talked about them as vague processes [*M* = 0.82, *SD* = 1.59; *t*(21) = 2.73, *p* < 0.02, *d* = 0.62].

**FIGURE 2:**
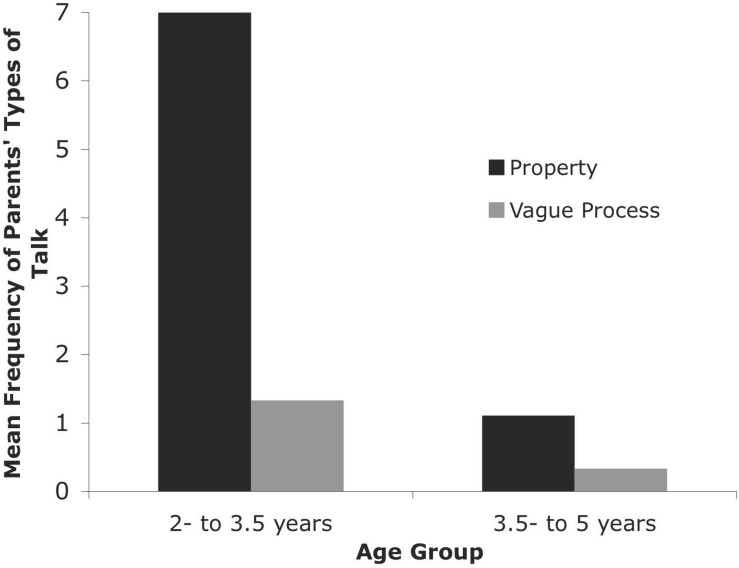
Parents’ Types of Talk for Younger and Older Children in the Gleason Database.

The 18 children whose ages were known were divided into two age groups: younger children who were 2–3.5 years old (four girls and five boys, mean age = 32 months, *SD* = 4.50) and older children who were 3.5–5 years old (three girls and six boys, mean age = 50.67 months, *SD* = 5.19). An analysis of how parents’ talk about heat and temperature varied with age was conducted in a 2 (type of talk: property, vague process) × 2 (age group: younger, older) mixed ANOVA on the frequency of the parents’ use of these types of talk. Type of talk was a within-subject factor and age group was a between-subjects factor. In a significant interaction [*F*(1, 16) = 7.00, *p* < 0.02, η*_*p*_*^2^ = 0.30], the parents of younger children used heat- and temperature-related words as properties of objects (*M* = 7.00, *SD* = 7.21) more than as vague processes [*M* = 1.33, *SD* = 2.18; *t*(8) = 3.18, *p* = 0.013, *d* = 1.06], whereas the parents of older children talked about properties (*M* = 1.11, *SD* = 1.96) and vague processes (*M* = 0.33, *SD* = 0.71) with similar frequency [*t*(8) = 1.58, *p* = 0.15] (see Figure 2).

The children’s use of the two types of talk was compared in a paired-samples *t*-test. The children talked about heat- and temperature-related words as properties (*M* = 1.00, *SD* = 1.66) more frequently than they talked about them as vague processes [*M* = 0.23, *SD* = 0.61; *t*(21) = 2.85, *p* < 0.01, *d* = 0.62]. In a separate mixed ANOVA on younger and older children’s talk, no age difference was found.

#### CHILDES Database: Hall

The parents’ use of the types of talk (property, substance, and process) was analyzed in a one-way repeated-measures ANOVA, revealing a significant type of talk effect [*F*(2, 37) = 17.29, *p* < 0.001, η*_*p*_*^2^ = 0.48], as shown in Figure 1. Pairwise comparisons indicated that parents talked about heat- and temperature-related words as properties (*M* = 4.95, *SD* = 5.37) more frequently than they talked about them as substances [*M* = 0.10, *SD* = 0.38; *t*(38) = 5.68, *p* < 0.001, *d* = 1.27] or as vague processes [*M* = 1.67, *SD* = 1.88; *t*(38) = 4.75, *p* < 0.001, *d* = 0.82] and that parents talked about heat- and temperature-related words as vague processes more frequently than as substances [*t*(38) = 5.09, *p* < 0.001, *d* = 1.16].

The categories of children’s talk were compared using one-way repeated-measures ANOVA. Type of talk was again significant [*F*(2, 37) = 24.27, *p* < 0.001, η*_*p*_*^2^ = 0.57]. Pairwise comparisons indicated that children talked about heat- and temperature-related words as properties (*M* = 5.77, *SD* = 5.25) more frequently than as substances [*M* = 0.10, *SD* = 0.38; *t*(38) = 6.87, *p* < 0.001, *d* = 1.52] or vague processes [*M* = 0.92, *SD* = 1.27; *t*(38) = 6.24, *p* < 0.001, *d* = 1.27] and that children talked about heat- and temperature-related words as vague processes more frequently than as substances [*t*(38) = 3.97, *p* < 0.01, *d* = 0.88]. Both parents and children had similar patterns of using property-, substance-, and vague process-based language with heat- and temperature-related words.

#### Snowman Study Data

As with the CHILDES data, we analyzed parents’ talk in the Snowman book-reading study with a 3 (type of talk: property, substance, vague process) × 4 (age: 2, 3, 4, 5-years-old) mixed ANOVA with type of talk as a within-subject factor and age as a between-subjects factor. The parents’ types of talk varied systematically [*F*(2, 46) = 49.89, *p* < 0.001, η*_*p*_*^2^ = 0.68], as shown in Figure 1. The parents talked about heat- and temperature-related words as properties (*M* = 4.64, SD = 3.55) more frequently than as substances [*M* = 0.11, *SD* = 0.38; *t*(50) = 9.12, *p* < 0.001, *d* = 2.15] or vague processes [*M* = 1.61, *SD* = 1.45; *t*(50) = 6.44, *p* < 0.001, *d* = 1.12] and as vague processes more frequently than as substances [*t*(50) = 7.08, *p* < 0.001, *d* = 1.42].

The children’s uses of these types of talk were analyzed in a 3 (type of talk: property, substance, vague process) × 4 (age: 2, 3, 4, 5-years-old) mixed ANOVA. A significant main effect of type of talk [*F*(2, 46) = 7.36, *p* < 0.01, η*_*p*_*^2^ = 0.24] was followed up by pairwise comparisons showing that children talked about heat- and temperature-related words as properties (*M* = 0.53, *SD* = 0.94) more frequently than as substances [*M* = 0.02, *SD* = 0.14; *t*(50) = 3.74 *p* = 0.001, *d* = 0.76] or as vague processes [*M* = 0.06, *SD* = 0.22; *t*(50) = 3.73, *p* < 0.01, *d* = 0.70]. Furthermore, a main effect of age [*F*(3, 48) = 4.44, *p* < 0.01, η*_*p*_*^2^ = 0.22] showed that 5-year-olds produced more heat- and temperature-related words (*M* = 0.46, SD = 0.31) than did 2-year-olds [*M* = 0.05, *SD* = 0.32; *t*(23) = 3.15, *p* < 0.02, *d* = 1.31] or 3-year-olds [*M* = 0.07, *SD* = 0.32; *t*(25) = 3.15, *p* < 0.02, *d* = 1.21], likely reflecting the older children’s greater productive vocabulary or contribution to conversations. The children’s types of talk did not vary by age group.

##### Vague process sub-codes

The parents in the Snowman Study also made some references to the three aspects of temperature change (proximity, time, and contact) (Parents and children in the CHILDES databases made references to these three aspects too infrequently to run analyses.) For example, a parent made reference to proximity by saying, “Cause you know what happens to snow when it gets *near* something hot?” Another parent made reference to contact by saying, “(eating ice cubes) makes him good and cold.” Some parents also made reference to time in the Snowman book, “And he goes out to play again. *Now* he’s all warm.” The higher frequency of these comments in the Snowman book may result from its focus on situations that are explicitly about temperature change and impending melting.

To examine parents’ talk about these three specific aspects of temperature change in the Snowman book study, a 3 (vague process sub-codes: proximity, time, contact) × 4 (age: 2-, 3-, 4-, 5-year-olds) mixed ANOVA was conducted on the frequency of parents’ use of these types of talk. Vague process sub-code was a within-subject factor and age was a between-subjects factor. In a main effect of vague process sub-codes [*F*(2, 46) = 4.57, *p* < 0.02, η*_*p*_*^2^ = 0.17], the parents made significantly more references to contact (*M* = 0.37, *SD* = 0.59) than to proximity [*M* = 0.10, *SD* = 0.36; *t*(50) = 2.64, *p* = 0.03, *d* = 0.55] or to time [*M* = 0.10, SD = 0.36; *t*(50) = 2.98 *p* < 0.02, *d* = 0.56]. There was no main effect of age and no interaction.

#### CHILDES Databases: Brown, Kuczaj, and Sachs

**FIGURE 3:**
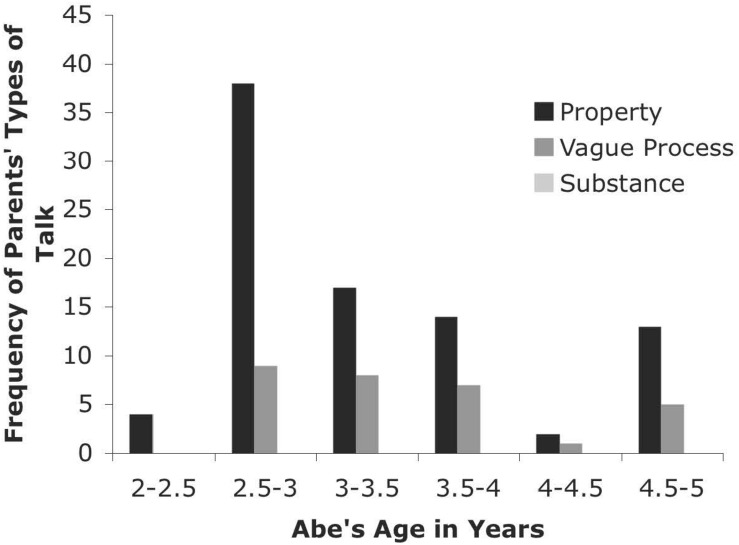
Types of Talk for Parents from CHILDES Abe Database.

**FIGURE 4:**
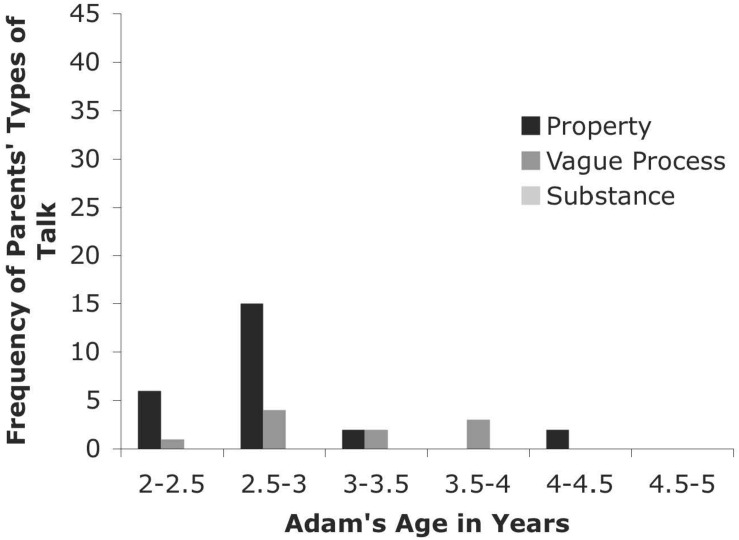
Types of Talk for Parents from CHILDES Adam Database.

**FIGURE 5:**
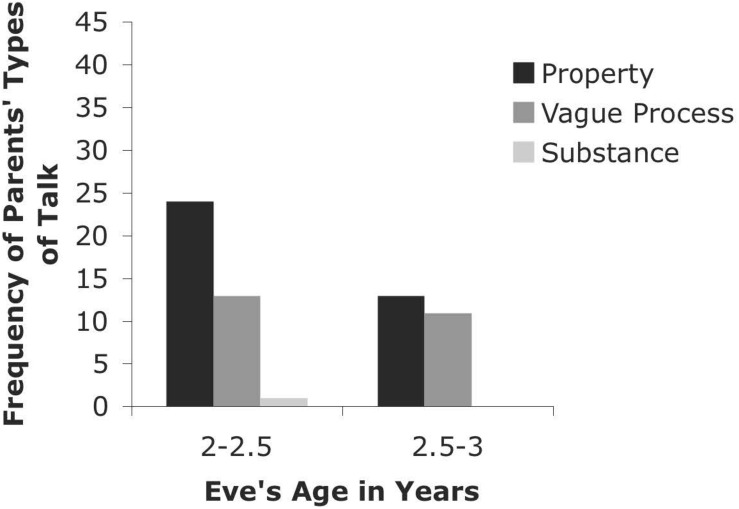
Types of Talk for Parents from CHILDES Eve Database.

**FIGURE 6:**
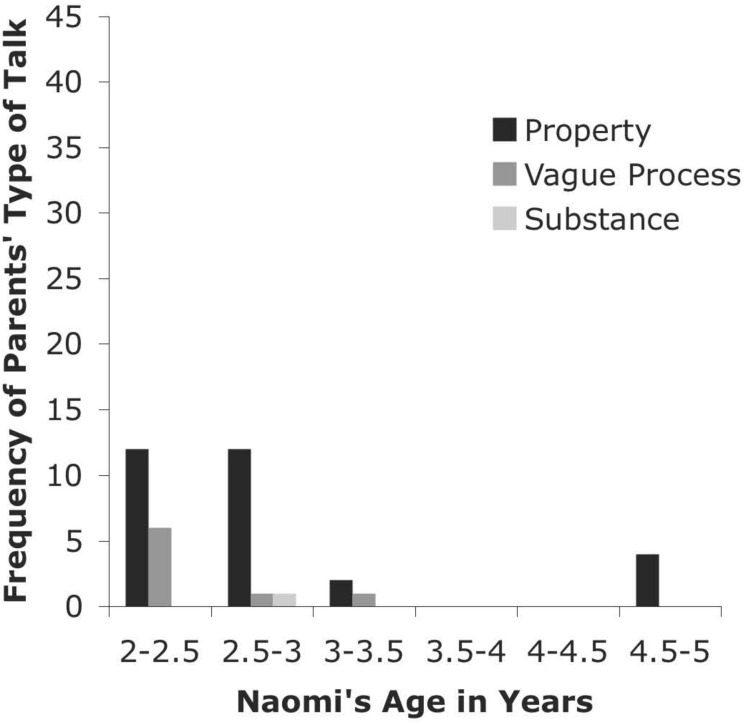
Types of Talk for Parents from CHILDES Naomi Database.

**FIGURE 7:**
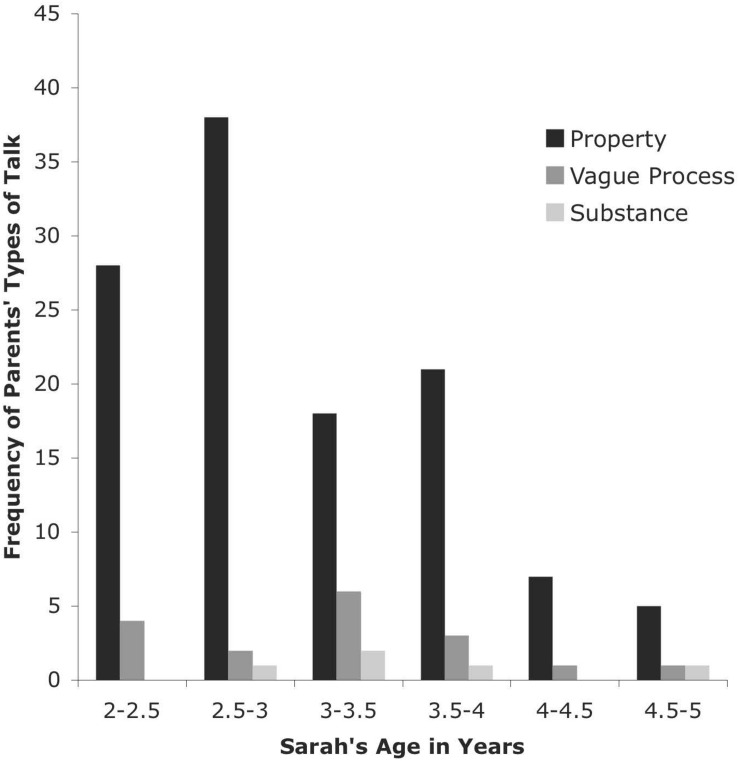
Types of Talk for Parents from CHILDES Sarah Database.

The longitudinal data from the five children in these databases (Abe, Adam, Eve, Naomi, and Sarah) are presented in Figures 3–7. The frequency with which the heat codes were observed in the parents’ speech is presented for each child by age grouping. The patterns in each longitudinal sample are case study examples that support the cross-sectional results. In general, the parents talked about these words as properties more often than as a substance or as a vague process. Children’s talk in these samples (although not depicted) followed a similar pattern.

In summary, across these three cross-sectional data sets and three longitudinal data sets, parents talked about heat- and temperature-related words as properties of objects more often than they talked about them as vague processes or as substances. A question to ask is whether this property-based talk might be related to a substance or a process view of heat. On the one hand, one could argue that thinking of heat as a substance might support the idea that heat is a property of hot objects. Parents in the Gleason Dinner database talked about temperature as property more with 2–3.5-year-olds but talked about properties and processes similarly with 3.5–5-year-olds. This finding is interesting in light of the evidence that young preschoolers are likely to answer heat- and temperature-related questions as if they believe that “hot” and “cold” are intrinsic properties of objects (e.g., Albert, 1978). On the other hand, one might argue that property talk could support a macro-level process view (although not a molecular emergent process view), depending on the real-world experiences within which children are hearing this language. In particular, if parents label objects that are not stable in their “hotness” or “coldness,” children might experience changes in objects’ temperature and see that the words “hot” and “cold” refer to transitory states. While this would still not be an emergent process view of heat at the molecular level, it could be different from the concept of heat as a substance.

As an exploratory analysis, we further coded all *property* statements made by parents as to whether they referred to items that generally have stable temperatures (including ice, fire, the sun, winter, freezers, etc.) or to items for which temperature is transitory (all other references). The percent agreement between two coders was 87% for 20% of parents’ property statements (Cohen’s Kappa = 0.66). Examples of properties coded as stable are as follows: “They’re getting in the freezer and the snowman liked it, ’cause it was nice and cold” and “The sun is hot, right.” Examples of properties coded as transitory are as follows: “Are you getting a little warm (referring to body temperature)?” “It’s very hot, honey (referring to coffee).”

We found that parents used property-based language for items that are transitory in their temperature (e.g., food) more often than for items that are stable in their temperature (e.g., ice), as shown in Table 5. This pattern was observed in all data sets except the Snowman book-reading where parents referred to objects of stable and transitory temperature with similar frequency, which is not surprising given the pictures of fire, ice, snow, and freezers in the book.

**TABLE 5:** Percentage of parents’ property-based talk that was sub-coded as references to objects with transitory or stable temperatures.

Database	Number of utterances	Transitory	Stable	Significance tests of means
**Group data sets**				
Snowman study	234	49%	39%	n.s.
Hall	187	84%	1%	*t*(39) = 5.41, *p* < 0.001, *d* = 1.20
Gleason dinner	76	76%	1%	*t*(16) = 2.64, *p* < 0.02, *d* = 0.94
**Individual data sets**				
Abe	86	84%	6%	
Adam	25	60%	8%	
Eve	40	75%	3%	
Naomi	53	68%	17%	
Sarah	125	83%	8%	

This pattern shows that the ways parents and children talk about heat and temperature likely relate to the activities they are engaged in, which is the focus of the next analysis.

### Analysis 3: Contexts for Talk About Heat and Temperature

Parents and children are not likely to engage regularly in formal school-like activities with heat and temperature; we next asked about the everyday situations where conversations about heat and temperature naturally arose in the CHILDES databases.

#### Main Finding 4

Emphasis on property, process, and substance in discussions of heat and temperature varied depending on the activity context. More specifically, conversations during meal times varied systematically, with more vague process talk while preparing meals and more property talk when eating.

Heat- and temperature-related words occurred most often during mealtimes and in conversations about weather and body temperature, as Table 6 shows. A descriptive analysis, displayed in Table 7, examined which types of talk (property, vague process, and substance) occurred in which types of contexts. Substance-based talk, although infrequent, occurred most often during meal times and conversations about the weather. Property-based talk also occurred most often during meal times and in conversations about the weather, with discussions of body temperature as the third most frequent category. Similarly, vague process talk occurred most often during meal times and in conversations about body temperature, perhaps because these situations relate to children’s direct experience with transitions in temperature. Furthermore, the context in which references to the word *heat* occurred most frequently was meal times, and most of these instances reflected the vague process use of the word heat (83%) versus the substance-involved-in-direct-process use (17%) [χ^2^(1, *N* = 12) = 5.33, *p* = 0.02].

**TABLE 6:** Contexts in which heat- and temperature-related talk occurred and the percentage of utterances that were coded as each type of context.

		Percentage of
Context	Description	Utterances
Meals	Food temperatures. Waiting for food to heat up or cool down.	37%
	Carefully eat hot foods.	
Weather	Temperature of outside/inside. The sun is hot.	17%
Body Temperature	People getting hot/warm/cold.	14%
Dressing	Putting on clothes to stay warm.	6%
	Taking off clothes to get cool.	
	Being too hot or cold wearing some type of clothing	
Touching Objects	Perceiving object temperatures. Getting burned.	4%
Heat Sources	Heat comes from certain objects. People or objects getting too close to heat sources	4%
	Reprimands for touching hot objects.	
Bathing	Taking a bath. Hand washing. Doing dishes.	4%
Swimming	Too cold to go swimming.	1%
Other	Using other meanings of the words.	14%
	Other specific situations such as “warming up” a vehicle.	
	Insufficient information present in the transcripts to determine the context.	

**TABLE 7:** Frequency of types of talk observed in each context (excluding “heat”).

	Property	Substance	Vague process	Other	Total
Dressing	72	3	34	2	111
Meals	480	10	117	17	624
Weather	249	13	28	3	293
Touching Objects	57	0	3	0	60
Bathing	65	1	5	1	72
Swimming	17	0	0	0	17
Body temp.	190	3	47	3	243
Heat sources	49	5	13	1	68
Other	87	12	14	124	237
Total	1266	47	261	151	1725

Meal times are likely to involve a number of different topics related to temperature, including both process talk (e.g., heating up and cooling down food) and property talk (e.g., discouraging someone from eating something that is too hot). As an exploratory follow-up coding, mealtime contexts were further divided into two categories – meal preparation and eating. The inter-coder reliability on 30% of the mealtime contexts was excellent (Cohen’s Kappa = 0.85). Of the 624 utterances coded as mealtime, 236 of them were reliably coded into the meal preparation and eating categories; the remaining 388 utterances could not be further distinguished as talk about eating versus preparing food (e.g., “It’s hot tapioca.”). Different patterns of property and vague process talk were found in the two contexts. Referring to the temperature-related words as properties occurred in 90% of the eating contexts versus only 33% of the meal preparation contexts. For example, in an eating context, the child said “This is not hot” and the mother replied “No, the lettuce is not hot.” In contrast, talking about these words as vague processes occurred in 67% of the meal preparation contexts and in only 10% of the eating contexts [χ^2^(1, *N* = 226) = 73.04, *p* < 0.001]. For example, in preparing to make candy, the child said, “OK now I think it’s finished just for one second” and the mother replied “No it needs to cool a little while longer.”

It seems that food preparation may facilitate more discussion of temperature change whereas conversation about eating food may facilitate more discussion of foods having the property of hot or cold. Food preparation activities seem to be a rich context for explicit conversation about the macro-level process of temperature change, beyond the more implicit transitory information that may be conveyed in parents’ talk about temperature as properties of objects. The everyday activities in which parents and children engage together seem to afford particular ways of talking about heat and temperature. Interestingly, many of the parents’ references to temperature drew upon the children’s direct experience with perceiving temperature in their bodies or the ways that temperature affects their interactions with food and other objects.

## General Discussion

The overall goal of this work is to characterize the “data” that children have available in conversations with others so that we can develop informed hypotheses about how these experiences relate to children’s developing reasoning about heat and temperature. We sought to describe the nature and the frequency of heat and temperature talk among parents and children. We uncovered a plethora of conversations about heat and temperature in a variety of activities. The naturalistic conversations that we sampled provided evidence of substance-based and process-based talk about *heat*. Both parents and children frequently discussed temperature-related concepts and temperature change. Thus, along with phenomenological experience, children seem to have access to an abundant amount of conversation about heat and temperature.

Part of the inspiration for this research was the intriguing speculation that students’ difficulties learning about heat result from their misconstrual of heat as a substance and that this misconstrual could result from hearing heat talked about as a substance in everyday life (Slotta and Chi, 2006). With the current data, we directly tested the latter claim and found that the young children in the sample actually rarely heard the word *heat*, but when parents did use the word they often did imply that heat is a substance or discussed heat as a process involving a substance, which supports Slotta and Chi (2006) claim. Not surprisingly, parents did not use the word *heat* in ways consistent with emergent processes. Despite these findings, there are three striking reasons why the overall picture does not align as clearly with Slotta and Chi’s substance-focused prediction as it might appear. First, the vast majority of conversations about heat and temperature involved not the word *heat* but other heat- and temperature-related words such as *hot* and *cold*, which most often functioned to indicate temperature as a property of objects (e.g., “The peas are hot.”), and the majority of these references described objects for which temperature is a transient property. Second, most parents and children also had a number of conversations about vague processes related to heat and temperature in certain activities. This leads to the third point: parents’ and children’s ways of talking about the concepts of heat and temperature varied in meaningful ways across a range of everyday situations, including meal times and getting dressed. We will discuss the implications of these findings below.

In this general discussion, we first discuss the results indicating that parents talk about heat and temperature as a property of objects as well as the findings that these conversations vary by context. Second, we discuss the potential implications of these conversations for children’s developing ideas about heat and temperature. Finally, we consider the potential theoretical implications of the findings.

### Property, Process, and Context

While parents’ mentions of heat were consistent with a substance view, more striking is the low frequency of opportunities to hear heat talked about at all. This supports Lautrey and Mazens (2004) prediction that there may be little support in everyday language for thinking about heat transmission (compared with concepts, although arguably esoteric, such as *resonate* for sound transmission). However, children did have ample opportunity to hear talk about temperature and temperature change. In particular, children in these families were often involved in conversations commenting on *hot* and *cold* as properties of objects. Many of these conversations were about objects that can and do change temperature. Only in the specific context of the Snowman book, with its focus on ice, fire, and snow, were objects with stable temperature talked about as often as objects whose temperature changes often.

Parents’ tendency to emphasize temperature as a property of objects varied systematically across contexts. For example, vague processes of temperature change were emphasized in food preparation contexts but properties were emphasized in eating contexts. It is not surprising that parents would emphasize the most relevant aspects of heat/temperature in each situation; for example, when a child is about to put food in her mouth, the end state of the heating process – hot peas – may be more relevant than how the peas became hot. While cooking, however, the process may be of most interest. This variability is consistent with diSessa’s notion that a variety of conceptualizations are available and chosen in the moment. Further empirical investigation should explore how children may be making sense of conversations about temperature that systematically vary by context.

While discussions of process were vague and not “scientific” and indeed likely reflect the parents’ own non-scientific views, there is evidence that children heard people talk about heat in ways that would direct them to attend to temperature change. In addition, future research and design of science curriculum could take into account the finding that children may be more accustomed to hearing about contact as relevant to heat, compared to other features. Parents in these samples offered information that heating and cooling are processes that relate to contact with sources of heat and less often to time and proximity. This should be taken into account in later teaching about thermodynamics in school. Understanding that contact is a relevant factor in heat transfer underlies the concept of conduction, which involves transfer of energy through matter. This could perhaps be easier for children than understanding that distance is a relevant factor in the process of heat transfer that underlies the concept of radiation, including the transfer of energy through air or across distances (e.g., the sun’s rays radiating to earth). It is true that the kinds of process talk parents used imply “direct processes” of heat transmission on a global level, which Slotta and Chi (2006, p. 263) identify as an inappropriate way to conceptualize the process of heat. While children are not experiencing support for thinking of heat as a molecular emergent process, they are hearing about heat as property and general process and not just as substance.

### Implications for Children’s Developing Ideas About Heat and Temperature

Because the goal of this work is to characterize children’s exposure to language “data” in the form of parents’ talk, these findings also result in a set of testable questions for future research regarding how children make use of this “testimony” from adults in developing their own concepts of heat and temperature. Although children infrequently heard parents talk about heat, we do not know for sure whether (and if so, how) these few instances contribute to children’s ideas. Children are likely to hear other non-entities (such as time or ideas, see Lakoff and Johnson, 1980) referred to as substances (e.g., “I ran out of time”; “That idea flew out of my mind”). Such talk could lead children to develop misconceptions (see Chi and Hausmann, 2003), however, it is possible that children may instead learn to recognize these figurative uses of language and learn that such language does not always imply substance. This view is consistent with Amin (2009, 2015) argument that children’s interpretations of such conceptual metaphors may be an important part of the process of conceptual change whereby embodied schemas are mapped to abstract concepts. It also seems possible that the abundant conversation about temperature and the paucity of conversation about heat may contribute to the conflation of the two concepts observed in prior research (Erickson, 1979; Wiser, 1995) and that when children begin to have formal instruction about “heat” they may try to make sense of the concept in relation to their abundant experience with conceptions of temperature as a property and temperature change.

The prevalence of conversation about *hot* and *cold* as properties is intriguing in light of the early research suggesting that young children (under age 9 years old) view heat and temperature as inherent properties of objects (e.g., Albert, 1978; Erickson, 1980). It is an open question whether these everyday conversations about hot or cold objects would lead children to expect hotness (or heat) and coldness to be inherent properties of objects. Contrary to this view, it seems relevant that we found much of the property-based talk in our samples to describe objects for which hot and cold are transitory properties. Future research is also needed to determine how conversation about hot and cold as properties may encourage substance or general process views of heat. Perhaps a notion of hotness as a property implies that a hot object contains more of the substance called *heat*. However, children’s experience with the changing temperature of familiar objects could also suggest to them that *heat* is rather a macro-level process of *heating*. For example, if adults comment on how “hot” the soup is and then a few minutes later tell the child “It’s OK to eat it now because it’s cool,” then one might expect this property talk to encourage a view of heating (or cooling) as a process. This example highlights that it is essential to consider these utterances within the meaningful activity contexts where they unfold. Finally, when parents discussed *hot* and *cold* as changing properties, they rarely elaborated on how things get hotter or colder. Perhaps the language that children hear could still be guiding them to look for explanations of the causes of temperature change (e.g., maybe “hot stuff” left the soup).

The current findings regarding the nature of everyday talk about heat and temperature may have implications for the interpretation of experimental data on children’s ideas of heat, hot, and cold. Questions used in earlier interview research, such as “Give me examples of heat” (Albert, 1978), may have been very confusing to young children; their answers may not have demonstrated their full understanding. Future research could explore whether children understand hot and cold differently for objects that are frequently involved in temperature change conversations versus those that are frequently involved in conversations focused on heat as a stable property (e.g., fire).

Parents’ and children’s tendency to focus on the hotness or the coolness of objects, and on the idea that changing from hot to cold (or cold to hot) is a process, perhaps reflects the everyday goals of ensuring safety when eating foods, deciding what to wear to go outside, and communicating one’s body temperature to obtain help in changing it. Using property or direct causal process language to describe the phenomena are perhaps useful ways to communicate about such goals in everyday situations. Although some argue that these kinds of talk are scientifically inaccurate, there seem to be important concepts explicitly and implicitly communicated through the vague process kinds of talk observed in our sample. The notion that everyday talk has overlapping but different goals from scientific talk is reminiscent of Vygotsky’s (1987) ideas about spontaneous and scientific concepts. He argued that everyday or “spontaneous” concepts may be incorrect from the perspective of school or “scientific” concepts, and yet children are likely to draw upon these early everyday conceptions as they try to make sense of the concepts that are presented in formal science instruction [see also diSessa (1993b)]. Thus, studies of children’s everyday knowledge could be informative for classroom science instruction (e.g., Moschkovich, 2002; Goldman, 2006; National Research Council, 2007). Moreover, scientists’ explanations of everyday phenomena regarding heat may have more in common with students’ explanations than one might think (Lewis and Linn, 1994), and these intuitive concepts should be taken seriously when thinking about formal science instruction (Hammer and Van Zee, 2006).

### Implications for Theory

We do not believe that these data support one theory over another. They are rather a systematic look at the actual linguistic information regarding heat and temperature available to young children, which various theories speculate about. The data are important in that they may indicate productive points of overlap between competing theories, and we suggest that a sociocultural perspective can help us attend to such overlap. In this section, we first speculate about how the data bear on the three main theories discussed earlier and, in conclusion, suggest points of overlap in attempts to move beyond pitting one theory against another.

#### Ontological Knowledge

While we found that not all talk about heat was substance-based, our findings generally support Slotta and Chi’s (2006) claim that everyday talk about heat does not overlap much with their conception of “scientific” talk about heat. The data clearly do not support an argument that parents teach children an emergent process way of thinking. On the other hand, the finding that everyday talk supports not just substance but property and vague process views of heat as well, opens up further questions regarding how parents’ talk may influence children’s developing everyday views of temperature and what the links as well as disconnects are between these everyday views and the later views learned in school. When considering development of reasoning about heat, an ontological view might further investigate the significance of the varied ways that children encounter different aspects of heat and temperature. The data show that children hear a lot of talk about temperature as properties of objects that can and do change in temperature frequently. How might children think differently about liquids versus solids, food versus non-edible objects, or the fluctuations in air temperature in a single day versus across a year with distinct seasons? How do ontological frames apply to the various situations that children encounter?

#### Theory Theory

Scholars who favor a theory theory approach are focused on the coherence of children’s thinking, regardless of whether it is “accurate” or similar to adult thinking (Carey, 1985). If children move from a conception of heat as a substance to heat as a process, then theory theory approaches would suggest that this is embedded in a larger intuitive theory change. If such a global theory change occurs, then our data on everyday ways of talking about heat would need to be taken into account as part of the picture of children’s changing conceptualizations of heat and temperature. How might children’s ideas be organized when thinking about different kinds of temperature-related phenomena? Might they have seemingly separate coherent theories about how air temperature changes versus how liquids heat up and cool down?

Another focus of theory theory is children’s sensitivity to the causal basis for events in the natural world (Gopnik, 2000). In some sense, the focus on causality in naïve theories could be seen as an additional barrier to learning the ontological category of emergent process because the type of causal thinking that is intuitive to people resembles direct causal processes. Children’s task in forming coherent theories of heat and temperature would involve integrating the various conceptualizations of temperature that they hear across many different contexts with their own experiences to construct a coherent view of these phenomena, including a mechanism for how temperature change works. Our data suggest that children hear little explicit discussion of what this causal mechanism might be and how it works. The fact that parents seem to be using multiple conceptual structures in the language that they use to describe heat, temperature, heating, and cooling likely makes the task of forming a coherent theory more difficult for children. This is perhaps exacerbated by the fact that the parents’ own theories of heat and temperature are based on intuitive views that are also likely inconsistent with the scientific view.

#### “Knowledge in Pieces”

From a “knowledge-in-pieces” view, the variation and the contradictions in the information available to children in family conversations might not be problematic for children’s developing understanding of heat and temperature because this is the way people are argued to think in general. In a recent study, for example, diSessa (2017) argues that the knowledge-in-pieces approach best explains the conceptual change regarding thermal equilibration observed in a high school classroom. By this approach, the language children hear in everyday conversations with their parents may offer children access to “pieces” of knowledge that they may later employ in different ways in different situations and would be consistent with studies showing that children’s answers to heat- and temperature-related questions vary depending upon the context and the method of assessment (Jones et al., 2000; Clark, 2006). In this view, the explanations used by parents and children in any given context are meaningfully related to and inseparable from those contexts because the explanations are also dependent upon the features of the situation, the goal of the activity, and the experience-based knowledge that each context can cue.

### Conclusion

Rather than attempting to decide among these dynamically changing theories, it may be more productive to find areas of agreement as we move forward to find a new framework. Including a sociocultural perspective may be useful in beginning to integrate the existing perspectives. A sociocultural view assumes that learning is situated and cannot be separated from the social and the cultural context (Rogoff, 1990; Lave and Wenger, 1991; Rogoff et al., 2018). Depending upon the particular context in which they are thinking, physicists and children may conceptualize heat in different ways (e.g., substance, direct causal process, emergent process) to accomplish the goals of the activity. Another point made by sociocultural theorists is that there is no pure way to measure underlying conceptions, and researchers should be wary about assuming that any particular assessment gives us the “true” measure of a person’s concept (Lave, 1997; Rogoff, 1997). Assessment can be viewed instead as documenting the ways that children participate in everyday activities and how such participation changes over time. Each experiment or naturalistic activity offers one glimpse of what children *do*, which may be more informative than trying to extract what concepts children *have*. From this stance, how children participate in everyday conversations is a necessary component of understanding the ways that their knowledge about heat and temperature is developing: *how* children’s knowledge develops is by participating in activities (Wertsch, 1979). A final relevant point from sociocultural theory is that attending to different aspects of sociocultural activity can allow for insight into varied ways of understanding learning and development (Rogoff, 1997). Applying this idea, a knowledge-in-pieces lens can help us understand how individuals are drawing upon a vast set of resources to reason in any given moment. These can include various modes of thinking, the language used to communicate ideas, and prior experiences with the phenomena itself and with communicating *about* the phenomena. A theory theory lens on the same behavior can help us understand how children’s thinking can appear to be organized consistently (or not) across contexts and how it can appear that consistency in reasoning changes over time. Rather than attributing the consistency to stable, coherent theories that persist across contexts, a sociocultural view would suggest that the consistency is related to the social contexts and kinds of situations that children reason in. The child-participating-in-context may be the consistency that is often attributed solely to the child. So we can then ask how children’s participation in reasoning activities changes over time instead of how children’s internal theories change.

Our goal is not to argue for a unitary direct causal path between parents’ speech and children’s reasoning about heat and temperature. We rather assume that children are active in constructing their understanding of the phenomena. We aimed to map out one crucial aspect of the terrain – the available resources in the everyday talk that children engage in and which they can draw upon in developing understandings about heat and temperature. The data that we sampled indicated that there is systematicity in the language that the children heard. The next steps involve investigating how children integrate the language that they hear with their physical and conceptual experiences involving heat and temperature.

Taking participation in social activity as a potential *mechanism* of cognitive development and learning, theoretical views should more fully take into account the everyday activities and uses of the terms *heat* and *temperature* in conversations and how children’s participation in such activities changes over time, especially when they encounter formal instruction. Clearly the present data support the notion that even very young children view heat and temperature as a topic with which they have much familiarity and their intuitive notions have been helpful to them as they navigate their physical world. We would argue that rather than focusing on replacing children’s experience-based knowledge (or “misconceptions”) with “correct” scientific knowledge, it may be more productive to consider the approach taken by diSessa and colleagues (Smith et al., 1993) as well as Wiser and Amin (2001) that there are different, valid ways of thinking about phenomena such as heat and temperature (scientific and everyday) and that learning can build on the intuitive notions. Even within theory theory approaches, the idea that inconsistent theories can co-exist in people’s minds has gained acceptance (Legare et al., 2012; Shtulman and Lombrozo, 2016). In support of this approach, educational researchers have theorized that meaningful learning can take place in “hybrid spaces” or “third spaces,” which are neither everyday nor school, where children are encouraged to use their everyday ideas to help them start to learn scientific concepts or dominant practices (Gutiérrez et al., 1999; Rosebery et al., 2005). For example, settings such as after-school programs and museums have been argued to be places where children may begin to learn to bridge their everyday and scientific views of concepts such as heat and temperature. Our study has demonstrated the importance of investigating young children’s everyday linguistic experiences in efforts to understand how such integrations of everyday and scientific thinking occur.

## Data Availability Statement

The datasets generated for this study are available on request to the corresponding author.

## Ethics Statement

The studies involving human participants were reviewed and approved by University of California Santa Cruz Institutional Review Board. Written informed consent to participate in this study was provided by the participants’ parents or legal guardians.

## Author Contributions

ML developed the conceptual motivation for the work, completed coding and statistical analysis for both the CHILDES study and the book-reading study, and wrote initial drafts of the manuscript. MC collaborated with two other researchers in designing the book-reading study and supervised data collection for that study, collaborated on the design of coding and analysis for both studies, and collaborated on writing and revising the manuscript. All authors contributed to the article and approved the submitted version.

## Conflict of Interest

The authors declare that the research was conducted in the absence of any commercial or financial relationships that could be construed as a potential conflict of interest.

## Footnotes

1 The term emergent process was introduced later; the early work referenced constraint-based interactions.

2 The substance-involved-in-direct-process coding category was applied to the heat- and temperature-related words that were originally coded as substance. This analysis of the few instances of temperature words used as if they were substances revealed that the coding category did not apply conceptually. For example, “He’s standing out in the cold” and “Because snowmen love the cold” seemed to reflect a substance-based conceptualization of coldness (the idea of coldness is a misconception in itself) but did not implicate cold(ness) in a process as was done with *heat*.
